# lncRNA-associated ceRNA network revealing the potential regulatory roles of ferroptosis and immune infiltration in Alzheimer’s disease

**DOI:** 10.3389/fnagi.2023.1105690

**Published:** 2023-02-16

**Authors:** Yejun Tan, Wang Tang, Wenbiao Xiao, Roujie Huang, Xin Li, Weijun Peng, Kuipo Yan, Yuan Cao, Yi Zeng, Jin Kang

**Affiliations:** ^1^Department of Rheumatology and Immunology, The Second Xiangya Hospital of Central South University, Changsha, Hunan, China; ^2^School of Mathematics, University of Minnesota Twin Cities, Minneapolis, MN, United States; ^3^Department of Geriatrics, The Second Xiangya Hospital of Central South University, Changsha, Hunan, China; ^4^Hunan Key Laboratory of Skin Cancer and Psoriasis, Hunan Engineering Research Center of Skin Health and Disease, Department of Dermatology, Xiangya Hospital, Central South University, Changsha, Hunan, China; ^5^Department of Integrated Traditional Chinese and Western Medicine, The Second Xiangya Hospital of Central South University, Changsha, Hunan, China; ^6^Department of Cardiology, The First Affiliated Hospital of Henan University of CM, Zhengzhou, Henan, China

**Keywords:** Alzheimer’s disease, ferroptosis, lncRNAs, ceRNA, immune infiltration, biomarker

## Abstract

**Background:**

Alzheimer’s disease (AD) is the most common form of dementia characterized by a prominent cognitive deterioration of sufficient magnitude to impair daily living. Increasing studies indicate that non-coding RNAs (ncRNAs) are involved in ferroptosis and AD progression. However, the role of ferroptosis-related ncRNAs in AD remains unexplored.

**Methods:**

We obtained the intersection of differentially expressed genes in GSE5281 (brain tissue expression profile of patients with AD) from the GEO database and ferroptosis-related genes (FRGs) from the ferrDb database. Least absolute shrinkage and selection operator model along with weighted gene co-expression network analysis screened for FRGs highly associated with AD.

**Results:**

A total of five FRGs were identified and further validated in GSE29378 (area under the curve = 0.877, 95% confidence interval = 0.794–0.960). A competing endogenous RNA (ceRNA) network of ferroptosis-related hub genes (*EPT1*, *KLHL24*, *LRRFIP1*, *CXCL2* and *CD44*) was subsequently constructed to explore the regulatory mechanism between hub genes, lncRNAs and miRNAs. Finally, CIBERSORT algorithms were used to unravel the immune cell infiltration landscape in AD and normal samples. M1 macrophages and mast cells were more infiltrated whereas memory B cells were less infiltrated in AD samples than in normal samples. Spearman’s correlation analysis revealed that LRRFIP1 was positively correlated with M1 macrophages (*r* = -0.340, *P* < 0.001) whereas ferroptosis-related lncRNAs were negatively correlated with immune cells, wherein miR7-3HG correlated with M1 macrophages and *NIFK-AS1*, *EMX2OS* and *VAC14-AS1* correlated with memory B cells (|*r*| > 0.3, *P* < 0.001).

**Conclusion:**

We constructed a novel ferroptosis-related signature model including mRNAs, miRNAs and lncRNAs, and characterized its association with immune infiltration in AD. The model provides novel ideas for the pathologic mechanism elucidation and targeted therapy development of AD.

## Introduction

Alzheimer’s disease (AD), the most common form of dementia, is characterized by significant cognitive deterioration, which impairs daily activities (Knopman et al., 2021). AD onset is typically marked by mild short-term memory impairment, followed by other hallmark symptoms, including deficits in language, visuospatial processing and executive functions (e.g., fine motor skill degradation; Derry et al., 2020). In 2021, aggregate payments for health care, long-term care and hospice services were estimated to reach $355 billion for individuals with AD or other dementias. The pathology of AD involves the accumulation of extracellular amyloid-β (Aβ) plaques in the cerebral cortex and intracellular tau-containing neurofibrillary tangles that occur sequentially in the locus coeruleus, entorhinal cortex, hippocampus, amygdala, temporal lobe, basal forebrain and isocortical association areas (Torok et al., 2018). Despite advancements in understanding the pathobiology of AD, current disease-modifying treatments are yet to cure AD. Hence, elucidating the underlying AD pathogenesis is vital, thereby driving research toward new therapeutic strategies.

Ferroptosis, an iron-dependent regulated cell death, occurs due to excessive lipid peroxidation (Stockwell et al., 2017). It has been implicated in the pathological neuronal death associated with various neurological diseases (including AD) (Stockwell et al., 2017) and support cell (i.e., supportive glial cells) damage, which consequently affects neurons in a wave-like propagation to trigger ferroptosis (Linkermann et al., 2014). Emerging evidence report that iron overload induces ferroptosis both *in vitro* and *in vivo* (Bao et al., 2021) by producing reactive oxygen species (Smith et al., 1997) and oligomeric tau, which is associated with ferroportin activity (Derry et al., 2020; Fasae et al., 2021). Moreover, high concentrations of iron in the insoluble Aβ plaques and neurofibrillary tangles are characteristics of AD (Fasae et al., 2021). Nonetheless, ferroptosis inhibitors and iron chelators (Wang et al., 2017) could specifically confer protection against ferroptosis, further mitigating neuronal oxidative damage and potentially delaying cognitive impairment. Glutathione peroxidase 4 (GPX4), a phospholipid peroxidase, plays a major role in protecting cells against ferroptosis by preventing excessive membrane lipid peroxidation. Both inactivations by inhibitors and GPX4 conditional deletion can precipitate ferroptosis, neuronal loss and astrogliosis, resulting in cognitive deficits and AD progression. Additionally, human studies report a strong association between age-related iron accumulation and AD clinical manifestation (Roberts et al., 2012; Hambright et al., 2017). Meanwhile, numerous pathological features of AD overlap with ferroptosis elements, pointing to a putative role for this novel form of cell death in AD pathogenesis. Therefore, the specific mechanism of ferroptosis in AD needs further exploration, for which bioinformatics analysis can provide preliminary directions.

Evidence has accumulated supporting that long non-coding RNAs (lncRNAs) play critical roles in gene regulation at the transcriptional and post-transcriptional levels (Statello et al., 2021). Additionally, lncRNAs are implicated in an array of physiological and pathological processes, including signal transduction and neuronal disorders (Shihabudeen Haider Ali et al., 2019). Notably, lncRNAs are abundant in the mammalian central nervous system (CNS), and links have been uncovered between the altered expression pattern of lncRNAs and the pathophysiology of AD (Wang et al., 2022). Marchese et al. (2017) report that the majority of lncRNAs play a role in the post-transcriptional cross-regulation of mRNA stability by operating as competing endogenous RNAs (ceRNAs) to compete for, and sequester shared microRNAs (miRNAs). These findings provide two directions for future research: ncRNAs as biomarkers and ncRNAs as therapeutic targets. Parallelly numerous ferroptosis-related genes (FRGs) have been identified as regulators and/or markers of ferroptosis, which could have promising therapeutic efficacy in ferroptosis-associated diseases (Shen et al., 2018; Chen et al., 2021; Zhang G. et al., 2021). Such as excess iron accumulation, elevated lipid peroxides, and glutathione peroxidase 4 (GPX4) levels. To date, few studies have reported on the role of lncRNAs in ferroptosis processes and the function of ferroptosis-associated lncRNAs in the context of AD. Therefore, it is of great significance to analyze the explicit regulatory networks of AD in the pathological process for advanced targeted therapeutics.

In this study, we aim to analyze the role of lncRNAs and immune cells in the pathogenesis of ferroptosis in AD using bioinformatics analysis. A lncRNA-associated ceRNA network containing five ferroptosis-related mRNAs and 17 differentially expressed lncRNAs (DElncRNAs) was constructed. Importantly, we further explored the potential regulatory mechanisms of this new network from the perspectives of ceRNA and immune infiltration in AD.

## Materials and methods

### Microarray data download and processing

**Figure 1:**
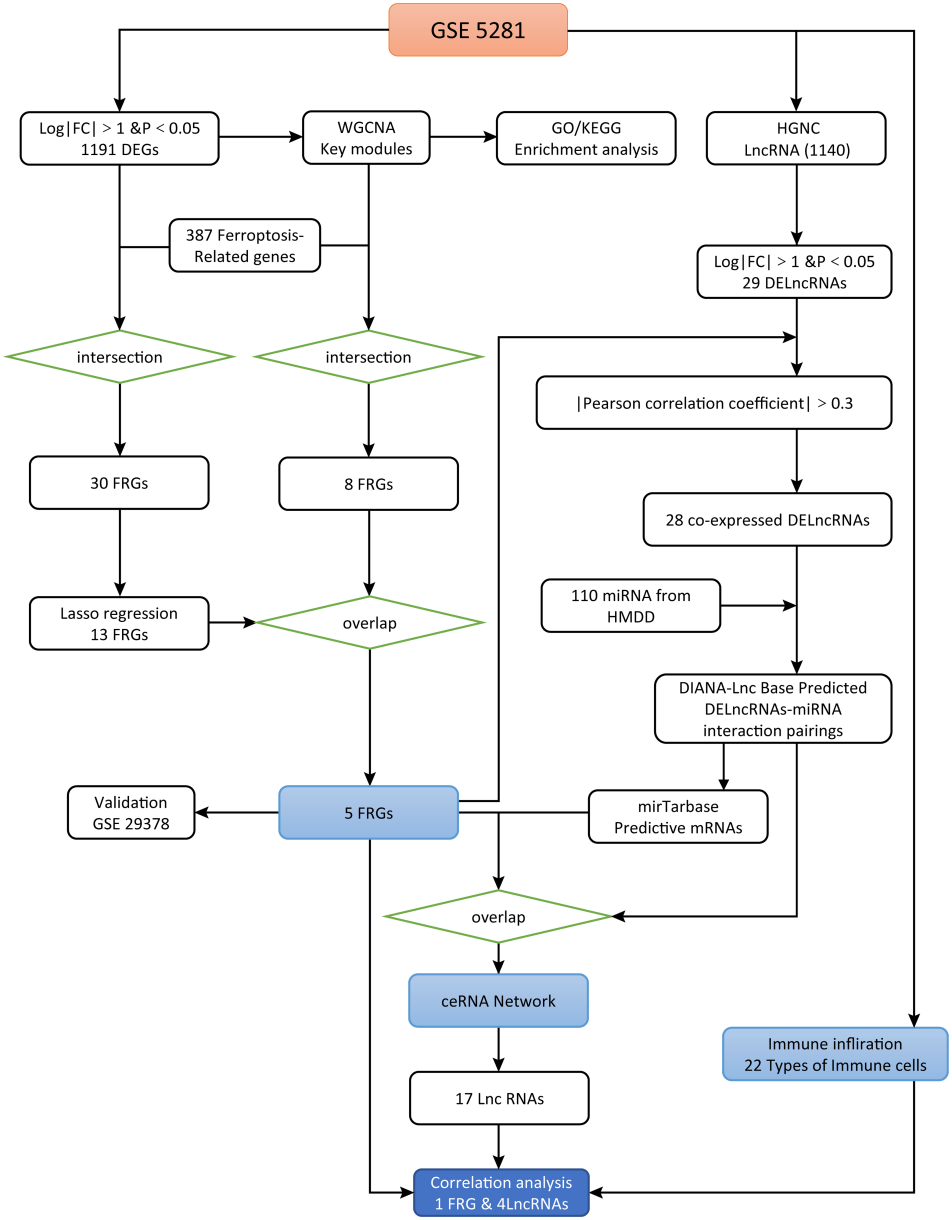
Study flowchart.

R language (version 4.11) was used for data analyses. Two microarray datasets of AD (GSE5281 and GSE29378) were downloaded from the GEO database^1^ (Liu et al., 2017). GSE5281 and GSE29378 are based on the GPL570 platform (Liang et al., 2007) and GPL6947 platform (Miller et al., 2013), respectively. Firstly, to explore the effects of these variables on the differential expression results, we corrected for their effects using linear regression model. In this process, we set brain region, gender, age as independent variables or covariates, and the gene expression level as the dependent variable. The residual derived from the linear regression value represent the expression value that correct the effect of these variables. To identify the FRGs and lncRNAs, we first analyzed data from the brain tissue of 87 AD and 74 normal samples (GSE5281). Subsequently, we validated the accuracy of these FRGs in distinguishing AD from normal samples in GSE29378 (brain tissue of 32 AD and 31 normal samples). The research flow chart is presented in Figure 1.

### Identification of DEGs

GSE5281 was downloaded from the GEO database using the ‘GEOquery’ package (Davis and Meltzer, 2007). Based on the introduction of GSE5281, individuals were stratified with respect to diagnostic groups (using both clinical and neuropathological criteria), age groups and APOE genotype. Additionally, tissue heterogeneity was eliminated prior to expression profiling by performing laser capture microscopy on all brain regions. We removed the probes corresponding to multiple molecules for one probe and only retained the probe with the largest signal value when encountering probes corresponding to the same molecule. The differential analysis between AD and the normal group was performed using the ‘*limma*’ software package. Furthermore, DEGs in microarray data analysis were defined as having *p* values <0.05, and |log (FC)| > 1 as the cutoff values. The *p*-value was used in our study to include more differential genes to input the data for further analysis. Furthermore, DEGs were visualized *via* volcano plot using the ‘*ggplot2*’ software package.

### FRGs in DEGs screened using LASSO regression

FerrDb is a ferroptosis database for the identification of ferroptosis-related markers, regulatory factors and ferroptosis-related diseases (Zhou and Bao, 2020). FRGs were downloaded from FerrDb. Following this, 387 FRGs overlapped with DEGs, thus revealing the FRGs in DEGs. The overlapping genes were visualized *via* Venn diagrams using the ‘*ggplot2*’ software package. Furthermore, we used LASSO to screen the overlapping FRGs in DEGs. As LASSO models have strong predictive values, we constructed a LASSO model to identify the best features of the high-dimensional data (Serra et al., 2019). The ‘*glment*’ software package was used to establish the LASSO model based on overlapping FRGs in DEGs, which could distinguish AD from the normal samples. The minimum lambda value was then used as a reference to determine the best variable in the model. Logistic regression analysis was performed on the genes obtained from the LASSO model to calculate the expression values and regression coefficients of overlapping FRGs in DEGs. The formula is as follows (Zhang T. et al., 2021):


$$\begin{array}{c} Index=ExpGene1\times Coef\;1+ExpGene2\times Coef\;2\\ +ExpGene3\times Coef\;3+\ldots \\ +ExpGeneN\times Coef\;N, \end{array}$$

‘Exp’ refers to the gene expression value, and ‘Coef’ refers to the gene regression coefficient.

### WGCNA identified FRGs in DEGs

The DEGs in GSE5281 were analyzed using WGCNA to obtain gene modules highly correlated with AD. The gene co-expression network was constructed using the R software package called ‘*WGCNA*’ (Langfelder and Horvath, 2008). We used the barplot function of R software to plot the eigengene barplots of grey and turquoise module genes to determine whether genes in the most related module have unique coherent expression patterns between disease and control. The adjacency matrix was constructed using a weighted correlation coefficient. Subsequently, the adjacency matrix was transformed into a topological overlap matrix (TOM). Then, the hierarchical clustering method identified the modules and calculated the characteristic genes. Finally, we assessed the correlation between the phenotype (i.e., disease state, brain region, gender and age) and each module using the Pearson correlation coefficient to determine the AD-related modules. The genes in these modules were considered to be AD-related module genes. The formula, which is similar to the expression matrix, is as follows (Zhang T. et al., 2021):


$$aij=0.5\times {\left[1+\mathrm{cor}\left(i,j\right)\right]}^{\beta }$$

Where *aij* is the adjacency function between gene *i* and gene *j*. To ensure the scale-free network, the soft threshold *β* value was 9, thus transforming the similarity matrix into an adjacency matrix. Subsequently, we constructed a TOM to measure the average network connectivity of each gene. We set relevant parameters and divided genes with similar expression profiles into different modules using dynamic tree cutting. Then, the hierarchical clustering method was used to construct the tree graph. Additionally, the correlation between the module characteristic genes and traits was calculated. Furthermore, these correlations were used to screen the module characteristic genes. Finally, the two gene modules with the strongest positive and negative correlation with AD were screened. The genes in these two modules were intersected with FRGs downloaded from FerrDb. We used the ‘*ggplot2*’ software package to draw Venn diagrams describing the overlapping genes.

### Identification and validation of FRGs

The FRGs in DEGs obtained from the LASSO model intersected with the FRGs in the WGCNA key modules, which were highly correlated with AD. Thus, overlapping FRGs were obtained and visualized in a Venn diagram using the ‘*ggplot2*’ software package. Subsequently, we used the R software package ‘*pROC*’ to plot receiver operating characteristic (ROC) curves and calculate the area under curves (AUCs) to evaluate the accuracy of these ferroptosis-related markers in distinguishing between AD and normal samples in the experimental set (GSE5281) and validation set (GSE29378) (Robin et al., 2011). In our study, ferroptosis-related markers could distinguish between AD and normal samples when AUC was higher than 0.7.

### Functional enrichment analysis

To better understand the function and pathway of genes in the WGCNA key modules (including FRGs), we conducted an enrichment analysis on genes in the key modules. In this study, the ‘*clusterProfiler*’ software package was used for enrichment analysis (Yu et al., 2012). Kyoto Encyclopedia of Genes and Genomes (KEGG) enrichment analysis was performed using the Enrichr tool to analyze the differentially expressed mRNAs (DEmRNAs) present in the key modules (Kanehisa and Goto, 2000; Kanehisa, 2019; Kanehisa et al., 2021). The main enriched pathways were visualized *via* a histogram using the ‘*ggplot2*’ software package.

### Identification of FRGs co-expressed lncRNAs

The HUGO gene named committee (HGNC) approved symbol of the lncRNA genes list was downloaded from https://www.genenames.org/ (Braschi et al., 2019). Based on previous studies, the list of lncRNA gene names was compared with the gene symbols in our dataset, and the overlapping genes were selected (Sabaie et al., 2021). The ‘*limma*’ software package was used to screen DElncRNAs, with |logFC| > 1 and adjusted *p-*values <0.05 as the cutoff values. We also used the ‘*ggplot2*’ software package to visualize DElncRNAs *via* a volcano plot. Pearson correlation coefficients between DElncRNAs and FRGs obtained from previous steps were calculated using the R software. lncRNAs with Pearson correlation coefficients higher than 0.3 or lower than –0.3 for at least one of the FRGs were considered correlated and co-expressed with FRGs(,). Finally, we used the ‘*ggalluvival*’ software package to visualize the co-expression between FRGs and DElncRNAs in the Sankey diagram.

### Construction of a ceRNA network of ferroptosis-related lncRNAs

DIANA-Lncbase V3 was used to identify experimentally validated interactions between miRNAs and lncRNAs (Karagkouni et al., 2019). ‘Homo sapiens species,’ ‘brain or peripheral blood tissues,’ and ‘high miRNA confidence levels’ were selected as criteria for DIANA-Lncbase queries. AD-related miRNAs were downloaded from the Human microRNA Disease Database (HMDD) V3.2 database (Karagkouni et al., 2019). After obtaining the interactions between miRNAs and DElncRNAs, the interactions between miRNAs and targeted mRNAs were predicted using strong experimental evidence from miRTarBase (Huang et al., 2020). Among the miRNAs obtained from miRTarBase, the miRNAs that could combine with targeted mRNA were selected for comparison with FRGs. Then, the overlapped mRNAs between the filtered targeted mRNA and ferroptosis-related markers were retained as the core of the ceRNA network. Finally, the ceRNA network was visualized using the Cytoscape software (Shannon et al., 2003).

### Immune infiltration

To better understand the relation between FRGs and lncRNAs and immune cells, we uploaded the expression profile data of 87 AD and 74 control samples (GSE5281) to CIBERSORT to obtain the immune cell infiltration matrix (Newman et al., 2015). Then, we used the ‘*Vioplot*’ software package to visualize the differences in immune cell infiltration between the AD and control groups. Additionally, R software was performed for the correlation analysis of FRGs and lncRNAs and 22 immune cell types. The ‘*ggplot2*’ software package was used to visualize the correlation between FRGs and lncRNAs and immune cells *via* lollipop plots.

### Ethics approval and consent to participate

Owing to the retrospective nature of this study, written informed consent was waived. As publicly available human databases were used in this study, we confirm that all methods were performed in accordance with the relevant guidelines and regulations.

## Results

### Identification of DEGs between the AD and normal groups

**Figure 2:**
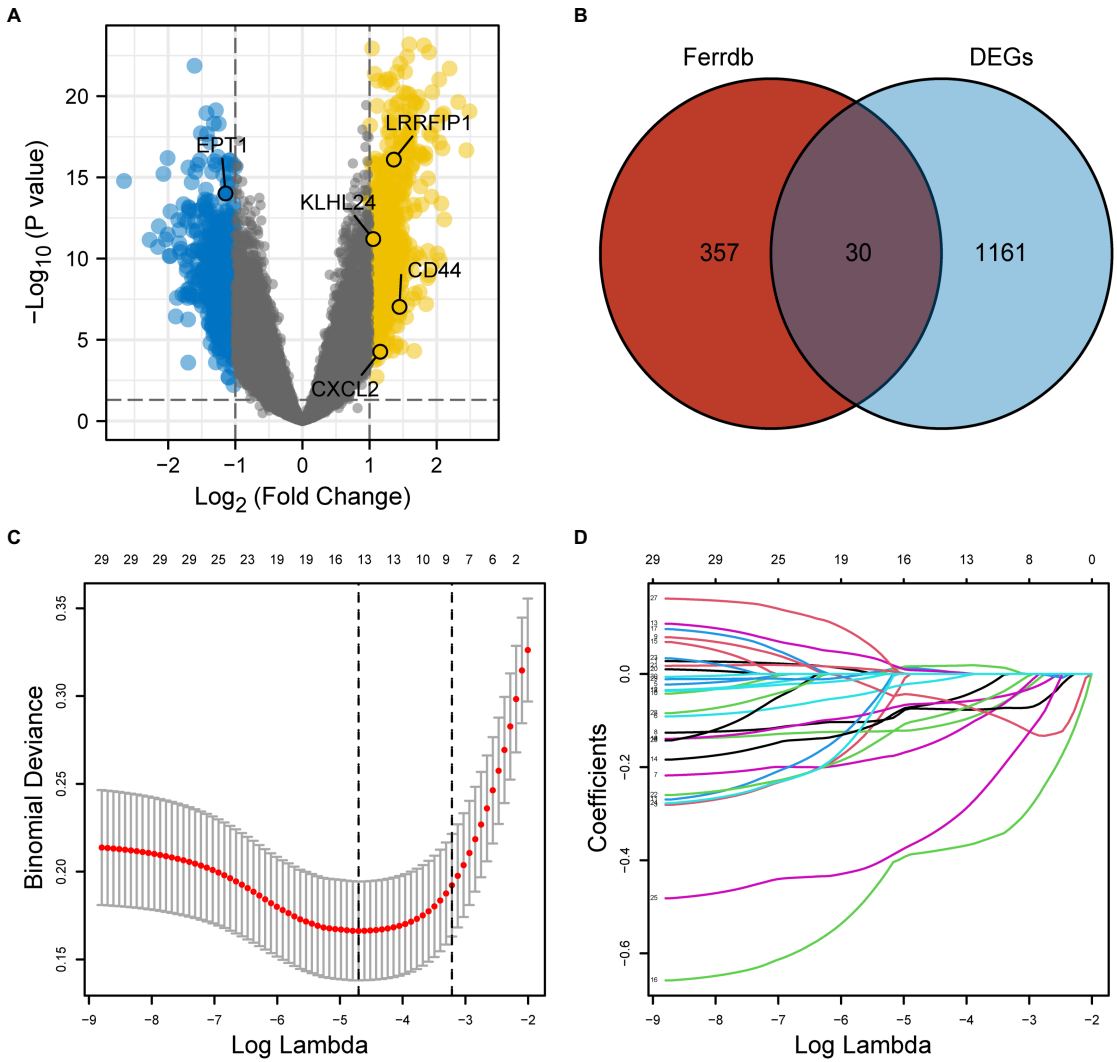
**(A)** Differentially expressed genes (DEGs) between the AD and control samples of GSE5281 (*p* < 0.05, with |log (FC)| > 1). **(B)** A total of 30 overlapping FRGs obtained from DEGs and 387 FRGs downloaded from FerrDb. **(C)** LASSO model. **(D)** The variation trajectory of each independent variable. DEGs, differentially expressed genes; FRGs, ferroptosis-related genes; AD, Alzheimer’s disease.

To identify differentially expressed genes between the AD and control groups, we first obtained the expression profile data of GSE5281 from the GEO database. We calculated the correlation between original and the residual expression level using Spearman correlation test, results showed a high degree of consistency between them (the median of the correlation coefficient is 0.93). Secondly, we calculated the expression difference between Alzheimer’s disease and normal samples using the corrected gene expression level. Among the 1,191 differentially expressed genes we detected, we found there was a high degree of consistency between the rank of gene expression difference derived from the original expression level and the rank of expression difference corrected by linear regression model (Spearman correlation coefficient = 0.82, *p* < 0.0001). Using *p* < 0.05 and |log (FC)| > 1 as the cut-off values, 1,191 DEGs were identified. These DEGs were visualized *via* a volcano plot (Figure 2A).

### FRGs in DEGs screened using the LASSO model

After the 387 FRGs downloaded from FerrDb were overlapped with DEGs, we obtained 30 overlapping FRGs (Figure 2B). The LASSO model was used to further screen the overlapping FRGs in DEGs (Figures 2C,D). A total of 13 FRGs (*CD44, CHMP6, CXCL2, DAZAP1, DDIT4, EPT1, GOT1, KLHL24, LAMP2, LRRFIP1MUC1, PPARA,* and *RB1*) were obtained from the LASSO model.

### WGCNA identified FRGs in DEGs

**Figure 3:**
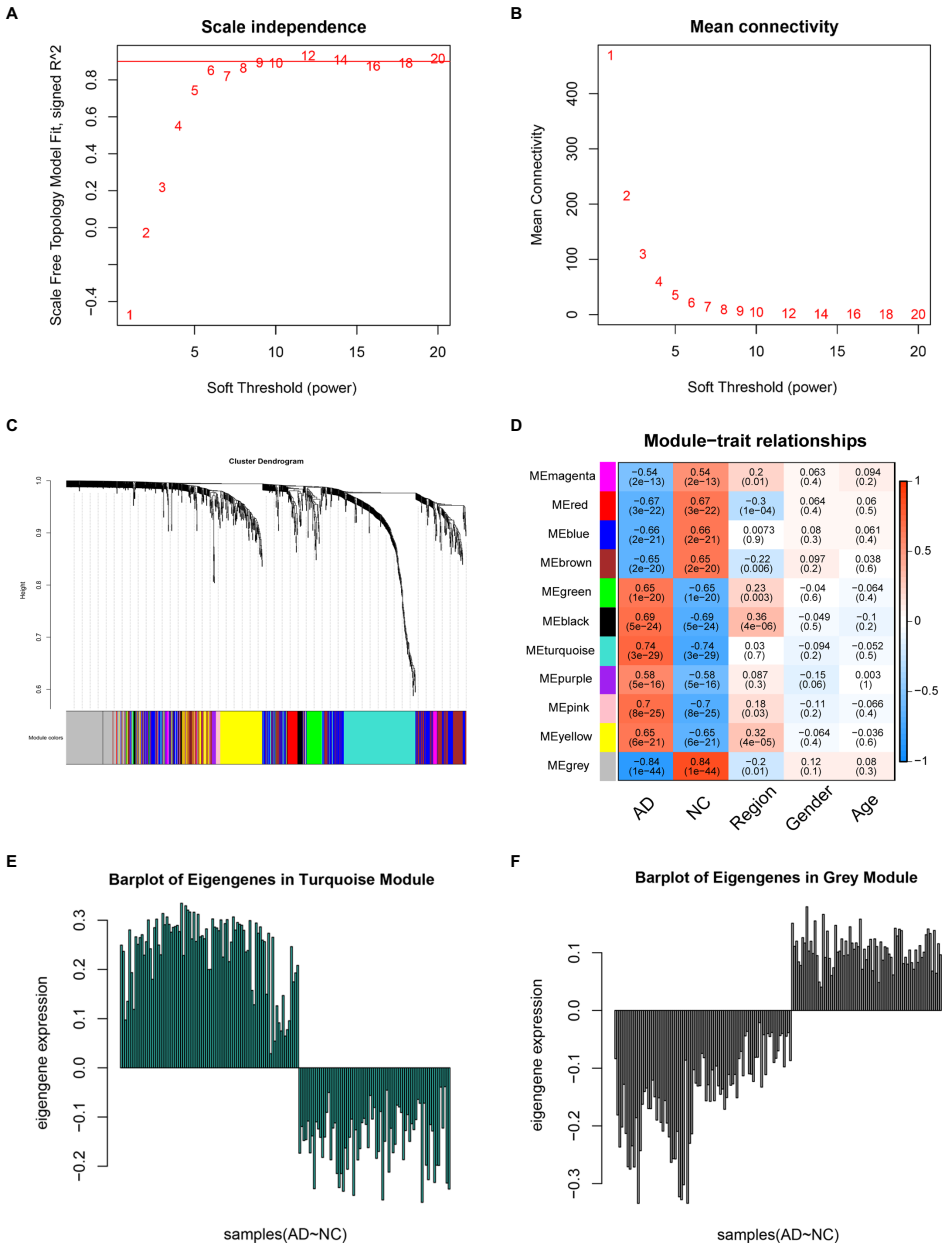
Weighted gene co-expression network analysis. **(A)** Analysis of the scale-free fit index for various soft-thresholding powers (*β*). **(B)** Analysis of the mean connectivity for various soft-thresholding powers. **(C)** Clustering dendrogram. **(D)** Module-trait associations as evaluated by correlations between module eigengenes and clinical traits; turquoise module represents a positive correlation with the clinical traits, whereas gray modules represents a negative correlation with the clinical trait. In each module, the top number represents the correlation and the bottom number represents the *p*-value. **(E)** Barplot of eigengenes in turquoise module. **(F)** Barplot of eigengenes in gray module.

The expression profiles of 1,191 DEGs from 161 samples were used to construct a weighted gene co-expression network (Figure 3). The key parameter related to a scale-free network is the soft threshold power value, which was set to 9 in this study to construct a scale-free network (Figures 3A,B). Then, the adjacency matrix and TOM were established, and the module characteristic genes representing the overall gene expression level of each module were calculated (Figure 3C). These genes were grouped into modules based on their correlations, identifying a total of 11 modules with a unique color (Figure 3D). Additionally, we analyzed the correlation between each trait gene and the phenotype (AD or control sample). Finally, the turquoise (Cor = 0.74, *p* = 3e-29) and grey modules (Cor = −0.84, *p* = 1e-44) were highly correlated with AD. Consequently, 246 genes positively related to AD in the blue module and 163 genes negatively related to AD in the brown module were selected for further analysis. Meanwhile, we used eigengene barplots which mapped the genes in grey and turquoise modules to confirm that genes in these two modules possess unique coherent expression patterns between disease and controlled states (Figures 3E,F).

### Identification and validation of FRGs

**Figure 4:**
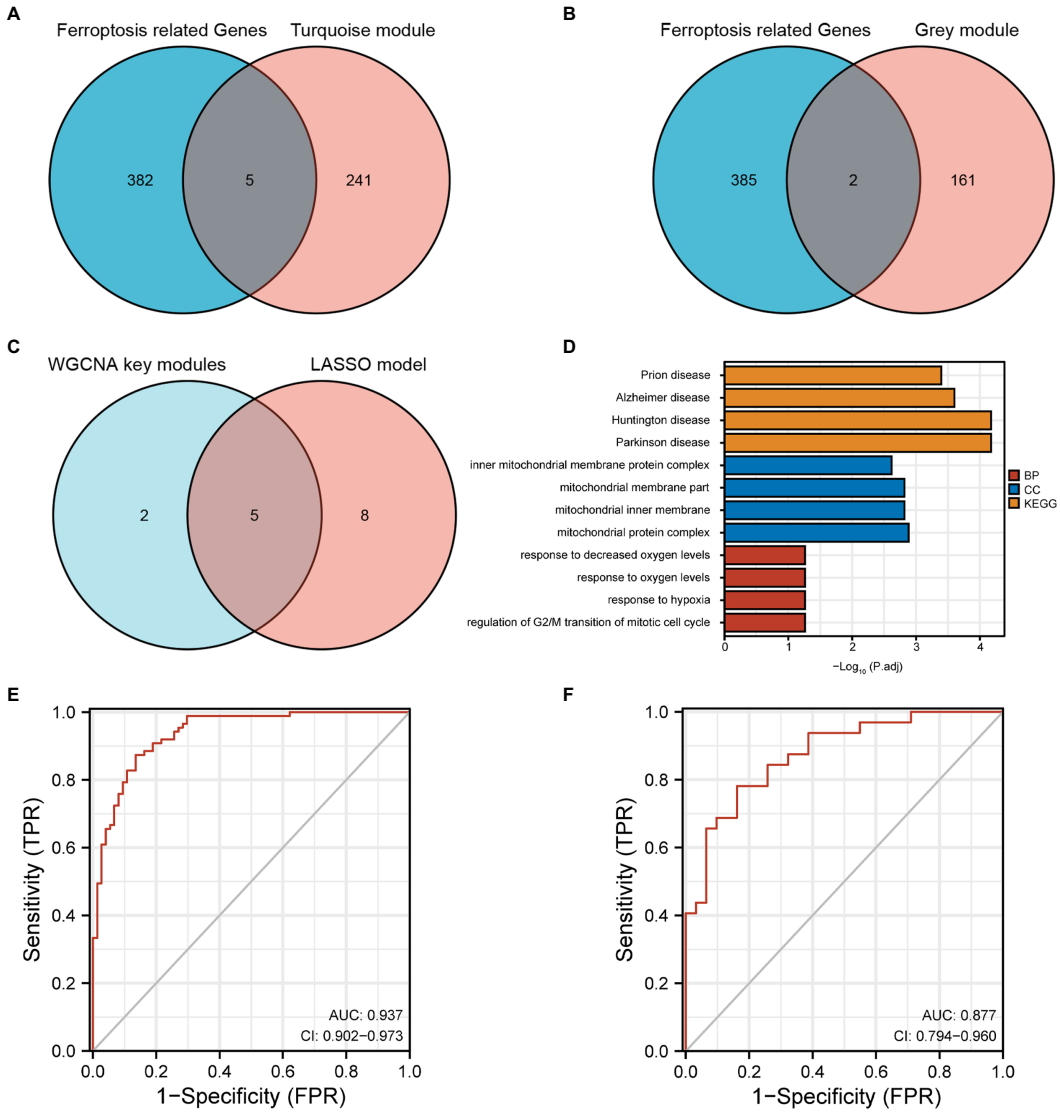
**(A)** Five FRGs obtained from the turquoise module. **(B)** Two FRGs obtained from the grey module. **(C)** Five FRGs from the intersection of the LASSO model and WGCNA key modules. **(D)** Main pathways of the genes from WGCNA key modules using functional enrichment analysis. **(E)** The ROC curve and the AUC of the five FRGs in GSE5281 (experimental set). **(F)** The ROC curve and the AUC of the five FRGs in GSE29378 (validation set). FRGs, ferroptosis-related genes; WGCNA, weighted gene co-expression network analysis; ROC, receiver operating characteristics; AUC, area under the curve; LASSO, least absolute shrinkage and selection operator.

A total of seven FRGs were obtained from turquoise and grey modules by the intersection of the genes in these two modules with the 387 FRGs downloaded from FerrDb (Figures 4A,B). The FRGs in DEGs obtained from the LASSO model intersected with the FRGs from the WGCNA key modules. Consequently, five FRGs (*EPT1*, *KLHL24*, *LRRFIP1*, *CXCL2*, and *CD44*) were obtained (Figure 4C). *EPT1* is a driver*, CD44* is a suppressor and the other genes were unclassified. Notably, these five FRGs showed high accuracy in distinguishing AD from the control in GSE5281 [AUC = 0.937, 95% confidence interval (95% CI) = 0.902–0.973] (Figure 4E). Subsequently, in the GSE29378 validation set, the five FRGs showed accuracy in distinguishing AD from control samples (AUC = 0.877, 95% CI = 0.794–0.960) (Figure 4F).

### Functional enrichment analysis

A total of 409 genes in WGCNA key modules were uploaded. Among these, 365 Entrez IDs were successfully converted. The conversion rate was 89%. Under the condition that *P*.adj < 0.05 and *q* value <0.2, 96 BP pathways, one CC pathway, eight MF pathways and four KEGG pathways were identified. The main pathways are visualized using a bar chart and presented in Figure 4D.

### Identification of the ferroptosis-related DELncRNAs

**Figure 5:**
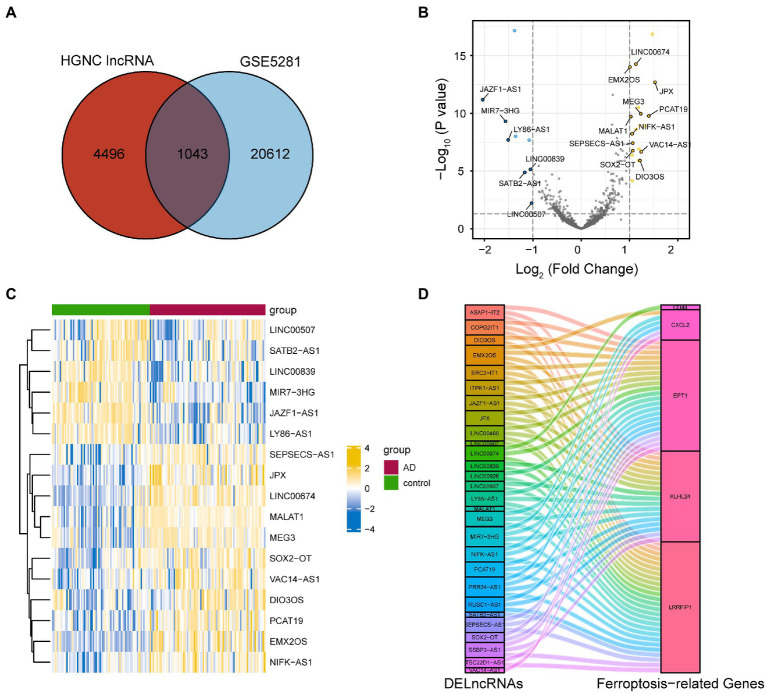
Identification of FRGs associated with differentially expressed lncRNAs. **(A)** A total of 1,043 lncRNA probes in GSE5281. **(B)** Differentially expressed lncRNAs in GSE5281. **(C)** The heat map of FRGs associated with differentially expressed lncRNA. **(D)** Co-expression network of differentially expressed lncRNAs and five FRGs. FRGs, ferroptosis-related genes; lncRNA, long non-coding RNA.

We downloaded the list of 5,540 lncRNAs that have been approved by HGNC. The lncRNA gene names were compared with the gene symbols in GSE5281, and the overlapping genes were obtained. We obtained 1,043 lncRNA probes in this dataset (Figure 5A). We also identified the DElncRNAs using the ‘*limma*’ software package, with |logFC| >1 and *P*. adj < 0.05 as the cut-off values. Consequently, a total of 29 DElncRNAs were obtained. A volcano plot was used to visualize the DElncRNAs (Figure 5B).

Furthermore, Pearson correlation coefficients between DElncRNAs and the five FRGs were calculated. Then, we removed the lncRNA without co-expression with a least one of the FRGs. The remaining 28 differentially expressed lncRNAs were correlated with at least one of the five FRGs (Pearson correlation coefficient higher than 0.3 or lower than –0.3). Finally, we used a heat map and Sankey diagrams to visualize the correlation between FRGs and DElncRNAs (Figures 5C,D).

### Construction of FRGs co-expressed lncRNA-related CeRNA network

**Figure 6:**
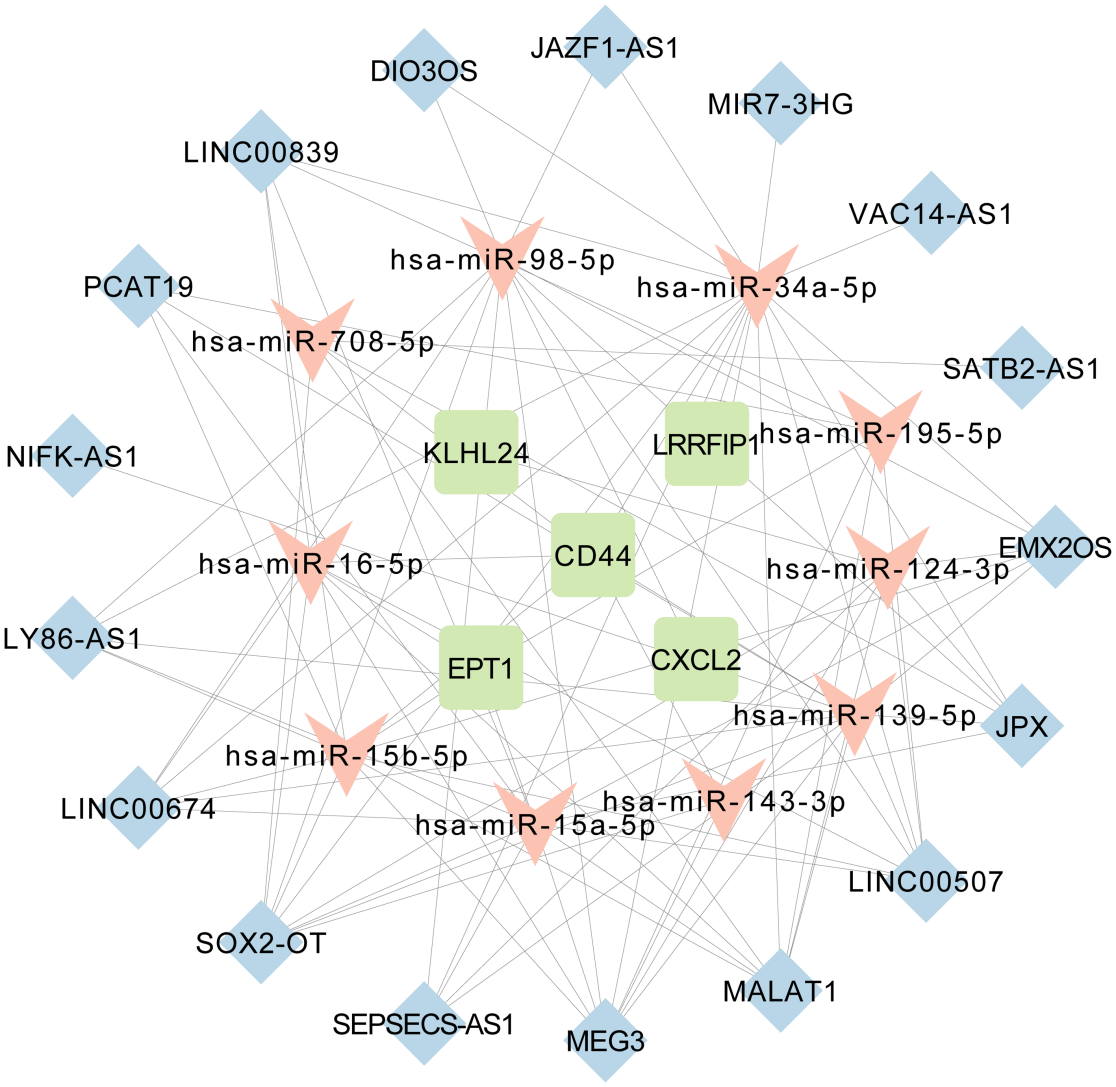
The long non-coding RNA-associated competing endogenous RNA (ceRNA) network of 17 lncRNAs, 10 miRNAs and five ferroptosis-related mRNAs.

DIANA-LNCBASE V3 predicted 35 miRNAs that could interact with 28 DElncRNAs among the 110 AD-related miRNAs downloaded from HMDD. miRTarBase predicted that these 35 miRNAs could interact with 7,815 target mRNAs. We combined these 7,815 predictive mRNAs with the five ferroptosis-related markers (*EPT1*, *KLHL24*, *LRRFIP1*, *CXCL2,* and *CD44*) as the core of the ceRNA network. Some of the DElncRNAs, targeted mRNAs and interacting miRNAs that were expressed in contrary patterns between lncRNAs and targeted mRNAs were deleted from the ceRNA network. Thus, we established a regulatory ceRNA network based on lncRNA-miRNA-mRNA interactions to explore the underlying mechanisms of ferroptosis in AD from a pathophysiological perspective. Finally, a ceRNA network with 32 nodes and 92 edges was constructed. Specifically, there were 17 lncRNA nodes corresponding to 10 miRNA nodes and five ferroptosis-related mRNA nodes. The 92 edges represent 79 lncRNA-miRNA and 13 miRNA-mRNA interacting edges (Figure 6).

### Immune infiltration

**Figure 7:**
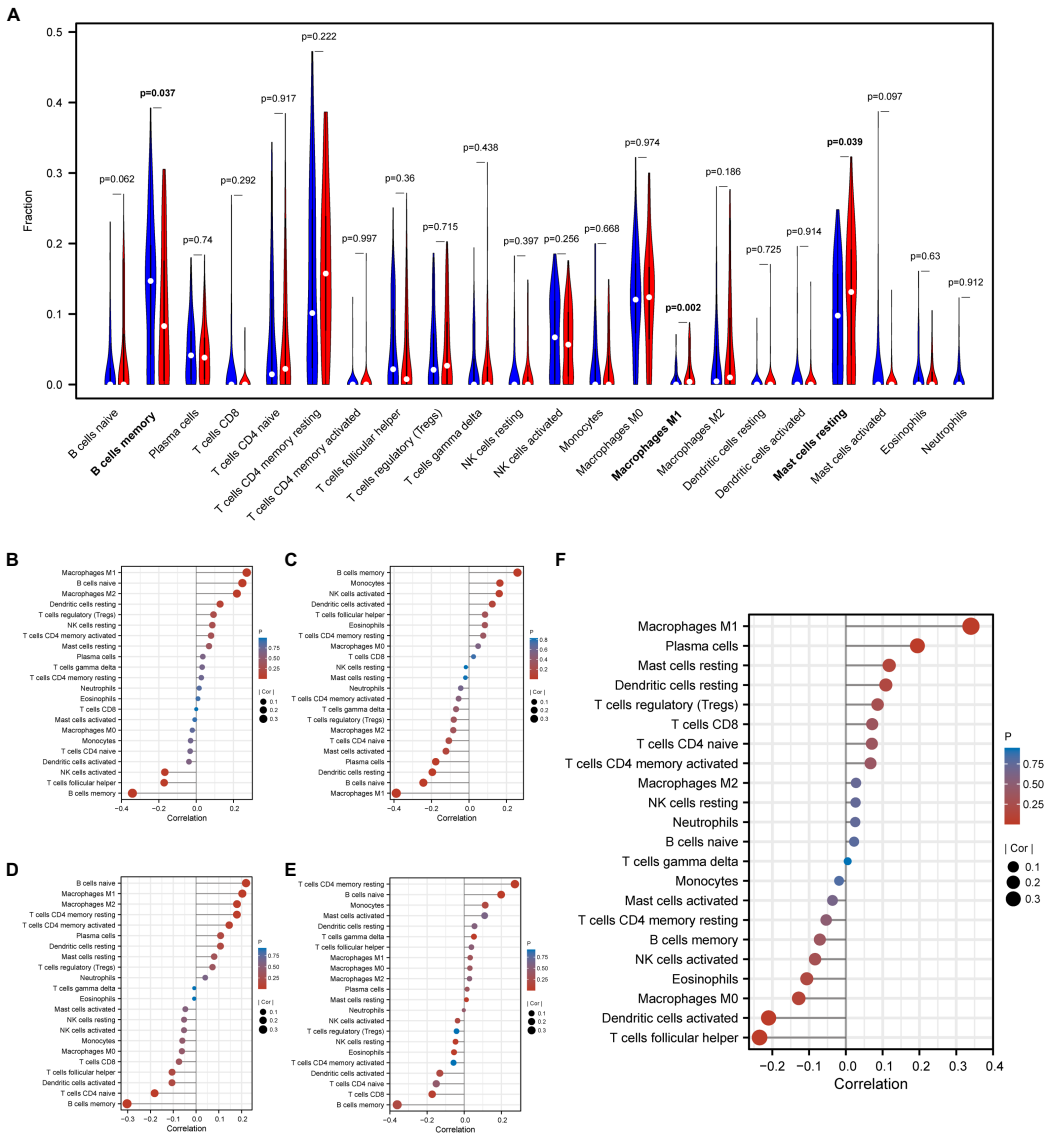
Evaluation and visualization of immune cell infiltration and the correlation between FRGs and lncRNAs and infiltrating immune cells. **(A)** Violin diagram of immune cell infiltration difference of 22 types of immune cells in AD and normal samples. **(B)** Correlation between EMX2OS and infiltrating immune cells. **(C)** Correlation between miR7-3HG and infiltrating immune cells. **(D)** Correlation between NIFK-AS1 and infiltrating immune cells. **(E)** Correlation between VAC14-AS1 and infiltrating immune cells. **(F)** Correlation between LRRFIP1 and infiltrating immune cells. FRGs, ferroptosis-related genes; lncRNAs, long non-coding RNAs; AD, Alzheimer’s disease.

CIBERSORT revealed the matrix of 22 types of immune cell infiltration. The violin plot of the immune cell infiltration difference showed that M1 macrophages and mast cells were more infiltrated in AD samples than in normal samples, while memory B cells were less infiltrated in AD samples (Figure 7A). Moreover, correlation analysis showed that FRG-*LRRFIP1* was positively correlated with M1 macrophages (*r =* −0.340, *p* < 0.001) (Figure 7F), whereas miR7-3HG was negatively correlated with M1 macrophages (*r =* −0.388*, p* < 0.001) (Figure 7C). Additionally, *NIFK-AS1*, *EMX2OS*, and *VAC14-AS1* were negatively correlated with memory B cells (*r* = −0.303, *p* < 0.001; *r* = −0.342, *p* < 0.001; *r* = −0.361, *p* < 0.001) (Figures 7B, D, E).

## Discussion

Ferroptosis is an iron-dependent programmed cell death that is morphologically and mechanistically distinctive from other forms of cell death. Recently, ferroptosis, characterized by iron accumulation and lipid peroxidation, has been linked to neurodegeneration and cognitive impairment (Dixon et al., 2012; Hambright et al., 2017). Consequently, the development of ferroptosis-based markers is gaining widespread interest, with investigations on iron chelation (Ashraf and So, 2020) and potential ferroptosis-associated therapy currently underway in a range of neurodegenerative disorders, including AD (Ficiarà et al., 2021). Meanwhile, increasing studies report that lncRNA plays an important role in the occurrence and development of AD (Zhang and Li, 2021; Zhang and Wang, 2021; Zhang J. et al., 2021). In this study, we attempted to elucidate the underlying mechanisms of ferroptosis in AD with respect to lncRNAs using bioinformatics.

We identified five differentially expressed FRGs through the intersection of the FRGs in WGCNA key modules and the LASSO model. Among them, *EPT1* was downregulated, while *KLHL24*, *LRRFIP1*, *CXCL2,* and *CD44* were up-regulated. *EPT1*, also termed selenoprotein I (SELENOI), is speculated to directly synthesize phosphatidylethanolamine that drives ferroptosis (Tavasoli et al., 2020). It also plays an indispensable role in myelination, neural development and maintaining phospholipid homeostasis in humans. This is consistent with our hypothesis that low *EPT1* expression could induce AD through ferroptosis. *LRRFIP1* has been overexpressed in both astrocytes exposed to ischemia and neurons that develop ferroptosis-induced cell death in response to pro-oxidant conditions (DeGregorio-Rocasolano et al., 2020). Studies report that *LRRFIP1* has a certain therapeutic effect on AD by inhibiting TNF-α signal transduction (Decourt et al., 2017). *GCF2*/*LRRFIP1*, which resides in the region-308 of the TNF-α gene promoter (Suriano et al., 2005), was recently reported to regulate pro-survival proteins and pathways in rat astrocytes, including β-catenin, Akt and mTOR signaling pathways (Gubern et al., 2014). Although its related research is still in its infancy, it has the potential as a therapeutic target for regulating TNF-α expression. In this study, *LRRFIP1* was predicted to be associated with ferroptosis, suggesting that *LRRFIP1* could induce neuronal ferroptosis by inhibiting TNF-α signaling. Thus, *LRRFIP1* has promising therapeutic potential for AD treatment. The inflammation-related gene *CD44* encodes a ubiquitously-expressed family of cell surface glycoproteins that have been associated with metastasis, inflammation and inflammation-induced neuronal damage (Pinner et al., 2017). A previous study showed that *CD44* expression was elevated in the lymphocytes of patients with AD compared with healthy controls (Uberti et al., 2010). Increased numbers of *CD44*-positive astrocytes have also been reported in the AD brain tissue (Akiyama et al., 1993). Furthermore, *CD44* lowers the vulnerability of cancer cells to oxidative stress and ferroptosis (Liu et al., 2019). As a member of inflammatory chemokines, *CXCL2* overexpression in AD could be related to cerebral vascular aging in patients with AD (Bryant et al., 2020). Combined with our study, it can be predicted that *CXCL2*-induced vascular aging in patients with AD could be associated with ferroptosis. Overall, these findings indicate that FRGs play a crucial part in the pathogenesis of AD.

Enrichment analysis linked differentially expressed FRGs to the regulation of the G2/M transition of the mitotic cell cycle, hypoxia response and mitochondrial activity. These biological processes are speculated to be the cause of AD. First, hypoxia has significant implications for AD pathogenesis by increasing Aβ accumulation, promoting tau hyperphosphorylation, disrupting the blood–brain barrier, inhibiting autophagic activity, exacerbating neuroinflammation and oxidation stress, inducing endoplasmic reticulum stress and leading to neurodegeneration. Second, mitochondrial dysfunction has been reported as a potential contributor to the etiology of AD. Several studies have demonstrated the presence of damaged mitochondria in brain tissues from sporadic and familial types of AD (Swerdlow, 2016). Microscopic features of AD include an increasing number of somatic mitochondrial DNA mutations; defective oxidative phosphorylation (Swerdlow, 2016); an imbalance between mitochondrial fission and fusion (Wang et al., 2009) and aberrant mitochondrial structure, motility and dynamics (Chen and Chan, 2009). Furthermore, tissue microarray analysis of biopsies from patients with AD revealed a substantial decrease in nuclear-and one mitochondrial-encoded subunit of the mitochondrial electron transport chain compared to age-matched controls. In terms of mitochondrial dysfunctions associated with AD (Desler et al., 2018), the mitochondrial capacity for oxidative phosphorylation is directly or indirectly inhibited in neurons and glial cells. Moreover, an inevitable outcome of impaired oxidative phosphorylation is the reduction in ATP production and, consequently, alterations in mitochondrial bioenergetics, which are necessary for the viability of affected diseased cells (Desler et al., 2012).

Furthermore, using multiple online databases, a ferroptosis-related lncRNA-associated ceRNA with five mRNAs, 28 lncRNAs and 110 miRNAs was constructed, which could reveal a new mechanism of interaction between RNAs. Additionally, these lncRNAs play crucial roles in multiple biological processes and AD development. LINC00507, first reported in the Mammalian Gene Collection Program, is expressed in non-human primates and humans in a cortex-specific manner (Ransohoff et al., 2018), indicating its involvement in the development of the cerebral cortex. Yan et al. report that the expression of LINC00507 was increased in the cerebral cortex and hippocampus of amyloid precursor protein/presenilin 1 (APP/PS1) mice, resulting in the activation of the p25/p35/GSK3β axis and tau pathology (Yan et al., 2020). However, it was dysregulated in the superior frontal gyrus of patients with AD in an age-dependent manner (Bosso et al., 1984). Moreover, it was downregulated in the current study. This difference could be attributed to the fact that all the brain regions as a whole were analyzed in the present study. miR7-3HG was significantly downregulated in the neurons of AD brains and exhibited a negative age-associated expression pattern (Cao et al., 2019), which is consistent with our findings. In addition to these age-associated lncRNAs, the expression of LY86-AS1, a gender-associated lncRNA, was negatively correlated with the Braak stage of AD in either females or males (Cao et al., 2019). MALAT1 has been demonstrated to play a neuroprotective role by reducing the levels of inflammation-associated miRNAs (Ma et al., 2019) and suppressing neuroinflammation (Masoumi et al., 2019). Ma et al. also reported that MALAT1 was reduced in Aβ1-42 treated cells, and it also decreased neuronal cell death (Ma et al., 2019). Mechanistically, MALAT1 binds directly to miR-124, a brain-enriched miRNA, and suppresses its expression. The neuroprotective effects of miR-124 have been proven in several CNS diseases (Sun et al., 2013), such as stroke. MEG3 is a highly neuro-specific lncRNA. Consistent with previous studies, MEG3 was highly upregulated in patients with AD in our study. Moreover, lncRNA MEG3 has been reported to activate neuronal necroptosis in AD. In the AD rat model, the intervention of MEG3 improves cognitive impairment, alleviates neuronal damage and inhibits astrocyte activation in hippocampus tissues in AD by inactivating the PI3K/Akt signaling pathway. MEG3, as an endogenous RNA of miR-181B, regulates the expression of ALOX15 in nerve cells in ischemic cerebral infarction (Yi et al., 2019). Arachidonic acid-dependent lipoxygenases (ALOXs) are the key enzymes that mediate lipid peroxidation and drive ferroptosis. Mechanistically, ALOXs oxidize PE-AA/ADA to PE-AA/ADA-OH to drive ferroptosis (Li et al., 2018). Thus, we established a regulatory ceRNA network based on lncRNA-miRNA-mRNA interactions to investigate the underlying mechanisms of ferroptosis in AD pathophysiology. These findings could provide some beneficial insights into AD treatment in clinical settings. For instance, many age-and gender-associated lncRNAs can be utilized in age-and gender-specific prevention and treatment options for patients with AD.

To better understand the relation between FRGs and lncRNAs and immune cells, we analyzed the infiltration of 22 types of immune cells in 87 AD and 74 normal samples. We found that M1 macrophages and mast cells were more infiltrated in AD samples than in normal samples. However, memory B cells were less infiltrated in AD samples, which is consistent with previous studies (Maslinska et al., 2007; Shaik-Dasthagirisaheb and Conti, 2016; Cao and Zheng, 2018; Munawara et al., 2021). Previous studies report that M1 macrophages, even though they are less abundant in the blood of patients with AD, are activated to produce a large number of pro-inflammatory cytokines. Moreover, M1 macrophages are also associated with the expression of classic inflammatory cytokines such as IL-1β and TNFα (Sudduth et al., 2013). Mast cells exist in the brain and peripheral blood of all mammalian species. The biological mediators of mast cells, including cytokines/chemokines, arachidonic acid products and stored enzymes, play important roles in AD (Shaik-Dasthagirisaheb and Conti, 2016). Mast cells can also be early detectors of amyloid peptides. According to a previous study, amyloid peptides activate the membrane Panx1 half-channel on mast cells, leading to threshing (Harcha et al., 2015). The oral tyrosine kinase inhibitor masitinib regulates mast cell threshing, differentiation and survival through C-kit and Lyn targeting (Dubreuil et al., 2009). A phase 2 randomized, placebo-controlled trial of masitinib as an adjunct therapy for patients with mild to moderate AD showed a reduced rate of cognitive decline over 24 weeks (Dubreuil et al., 2009). Regarding memory B cells, during aging, the diversity of B cell receptor libraries and the conversion of memory B cells are significantly reduced, which is accompanied by a diminished antibody response to antigen attack. Conversely, a unique subpopulation of mature B cells, called age-related B cells, accumulate in elderly mice and the older human population, which could promote inflammation and autoimmunity while inhibiting B-cell lymphogenesis (Naradikian et al., 2016; Riley et al., 2017). However, the function of B cells in AD remains to be explored further.

Furthermore, we also analyzed the correlation between immune cells and FRGs and lncRNAs. Correlation analysis showed that FRG-LRRFIP1 was positively correlated with M1 macrophages, whereas miR7-3HG was negatively correlated with M1 macrophages. Additionally, *NIFK-AS1* was negatively correlated with memory B cells. As mentioned above, *LRRFIP1* could have a certain therapeutic effect on AD by inhibiting TNF-α signaling whereas M1 macrophages are associated with the expression of classic inflammatory cytokines, such as IL-1β and TNFα (DeGregorio-Rocasolano et al., 2020). Therefore, the role of *LRRFIP1* as an FRG in the pathological process of AD deserves further investigation. Previous studies report that the expression of age-negatively correlated lncRNA miR7-3HG is significantly reduced in the AD brain compared with age-matched normal brains (Cao et al., 2019). However, reports on the relationship between miR7-3HG and M1 macrophages are scarce. As a result, the pathological relationship between miR7-3HG and AD needs further study. *NIFK-AS1* was reported to be correlated with M2 macrophages in endometrial cancer (Zhou et al., 2018). However, the correlation between *NIFK-AS1* and memory B cells in AD has not been reported so far, to our best knowledge, thereby requiring further study. *EMX2OS* is an antisense transcript of EMX2. Key genes in brain development and cortical regionalization, such as the Empty Spiracle Homeobox 1 and 2 (EMX1 and EMX2) genes, are abnormally methylated in neurodegenerative diseases (Muzio and Mallamaci, 2003). However, the correlation between *EMX2OS* and memory B cells remains unexplored, requiring further exploration.

Despite selecting a dataset with a large sample size, several limitations exist. First, it is a retrospective analysis with inherent biases, and further prospective investigations are needed to corroborate the findings. Second, *in vitro* and *in vivo* validation using experimental or clinical samples is required for the identified FRGs and lncRNAs. Third, no additional grouping was made for analysis owing to data constraints.

## Conclusion

In summary, this study constructed a novel ferroptosis-related signature model including mRNAs, miRNAs, and lncRNAs, and characterized its association with immune infiltration in Alzheimer’s disease. This model provides a new perspective to explain the internal relationship between ferroptosis and lncRNA in Alzheimer’s disease. Meanwhile, the model provides a new idea for the pathologic mechanism as well as targeted therapies of AD.

## Data availability statement

The original contributions presented in the study are included in the article/supplementary material, further inquiries can be directed to the corresponding authors.

## Author contributions

JK and YT designed the study concept. YT, WT, and WX analyzed and interpreted the data, drafted and revised the manuscript. YT guided the R analyses. RH, XL, and YC performed the Cytoscape analyses and supervised the study process. WP, YZ, and KY participated in reviewing and obtained the funding. All authors read and approved the final manuscript.

## Funding

This research was supported by the Science and Technology Program of Henan, China (Nos. 192102310161 and 182102310291), the National Science & Technology Fundamental Resources Investigation Program of China (No. 2018FY100900), the National Natural Science Foundation of China (No. 815771151), the Hunan Provincial Natural Science Foundation of China (No. 2021JJ30923), the National Natural Science Foundation of China (No. 82201614), and the Scientific Research Launch Project for new employees of the Second Xiangya Hospital of Central South University.

## Conflict of interest

The authors declare that the research was conducted in the absence of any commercial or financial relationships that could be construed as a potential conflict of interest.

The reviewer JG declared a shared affiliation with the authors YT, JK, YZ, WT, RH, WX, YC, XL, and WP to the handling editor at the time of review.

## Publisher’s note

All claims expressed in this article are solely those of the authors and do not necessarily represent those of their affiliated organizations, or those of the publisher, the editors and the reviewers. Any product that may be evaluated in this article, or claim that may be made by its manufacturer, is not guaranteed or endorsed by the publisher.

## Footnotes

^1^ http://www.ncbi.nlm.nih.gov/geo
